# Crystal structure of bis­{*N*-[2-(di­methyl­amino)­eth­yl]quinolin-8-amine-κ^3^ *N*,*N*′,*N*′′}nickel(II) dichloride 3.5-hydrate

**DOI:** 10.1107/S1600536814019035

**Published:** 2014-08-30

**Authors:** Benson M. Kariuki, Abdul-Razak H. Al-Sudani

**Affiliations:** a School of Chemistry, Cardiff University, Main Building, Park Place, Cardiff CF10 3AT, Wales; b Department of Chemistry, College of Science, Baghdad University for Women, Baghdad, Iraq

**Keywords:** crystal structure, *N*-[2-(di­methyl­amino)­eth­yl]quinolin-8-amine, nickel(II) complex, hydrogen bonding

## Abstract

In the title compound, [Ni(C_13_H_17_N_3_)_2_]Cl_2_·3.5H_2_O, the geom­etry of the NiN_6_ complex cation is slightly distorted octa­hedral, with a facial arrangement of the two tridentate *N*-[2-(di­­methyl­amino)­eth­yl]quinolin-8-amine ligands around the metal ion. The asymmetric unit consists of two independent complex half-mol­ecules located on centres of inversion, together with two chloride counter-anions and 3.5 water mol­ecules of solvation, one of which is disordered across an inversion centre. In the crystal, O—H⋯O, O—H⋯Cl and N—H⋯Cl hydrogen-bonding inter­actions form a three-dimensional network structure.

## Related literature   

For background to N-containing ligands, including quinoline derivatives, see: Kizirian (2008 ▶); Miodragovic *et al.* (2008 ▶); Puviarasan *et al.* (2004 ▶); Singh *et al.* (2008 ▶); Zhang *et al.* (2009 ▶). For complexes incorporating *N*-[2-(di­methyl­amino)­eth­yl]quinolin-8-amine, see: Al-Sudani & Kariuki (2013 ▶); Al-Sudani (2014 ▶).
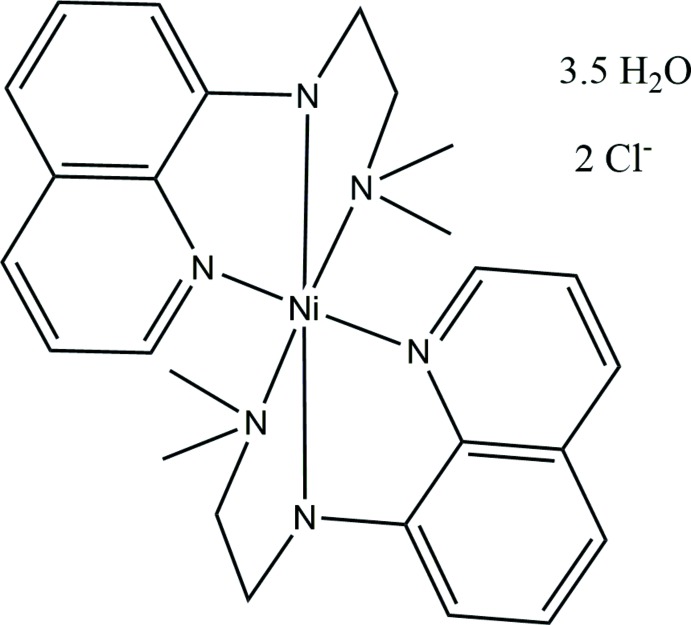


## Experimental   

### Crystal data  

[Ni(C_13_H_17_N_3_)_2_]Cl_2_·3.5H_2_O
*M* *_r_* = 623.26
Triclinic, 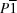

*a* = 10.6940 (2) Å
*b* = 11.8612 (4) Å
*c* = 12.1088 (3) Å
α = 90.520 (1)°
β = 101.181 (2)°
γ = 102.259 (2)°
*V* = 1470.39 (7) Å^3^
*Z* = 2
Mo *K*α radiation
μ = 0.88 mm^−1^
*T* = 150 K
0.18 × 0.16 × 0.08 mm

### Data collection  

Nonius KappaCCD diffractometer
Absorption correction: multi-scan (*DENZO*/*SCALEPACK*; Otwinowski & Minor, 1997 ▶) *T*_min_ = 0.857, *T*_max_ = 0.933
10258 measured reflections
6714 independent reflections
5592 reflections with *I* > 2σ(*I*)
*R*_int_ = 0.023

### Refinement  

*R*[*F* ^2^ > 2σ(*F* ^2^)] = 0.039
*wR*(*F* ^2^) = 0.090
*S* = 1.03
6714 reflections
383 parameters
12 restraints
H atoms treated by a mixture of independent and constrained refinement
Δρ_max_ = 0.46 e Å^−3^
Δρ_min_ = −0.59 e Å^−3^

### 

Data collection: *COLLECT* (Nonius, 2000 ▶); cell refinement: *SCALEPACK* (Otwinowski & Minor, 1997 ▶); data reduction: *DENZO* (Otwinowski & Minor, 1997 ▶) and *SCALEPACK*; program(s) used to solve structure: *SIR92* (Altomare *et al.*, 1993 ▶); program(s) used to refine structure: *SHELXL2013* (Sheldrick, 2008 ▶); molecular graphics: *Mercury* (Macrae *et al.*, 2008 ▶), *ORTEP-3 for Windows* (Farrugia, 2012 ▶) and *CHEMDRAW Ultra* (Cambridge Soft, 2001 ▶); software used to prepare material for publication: *publCIF* (Westrip, 2010 ▶).

## Supplementary Material

Crystal structure: contains datablock(s) I, New_Global_Publ_Block. DOI: 10.1107/S1600536814019035/zs2312sup1.cif

Structure factors: contains datablock(s) I. DOI: 10.1107/S1600536814019035/zs2312Isup2.hkl

**Fig. 1.**
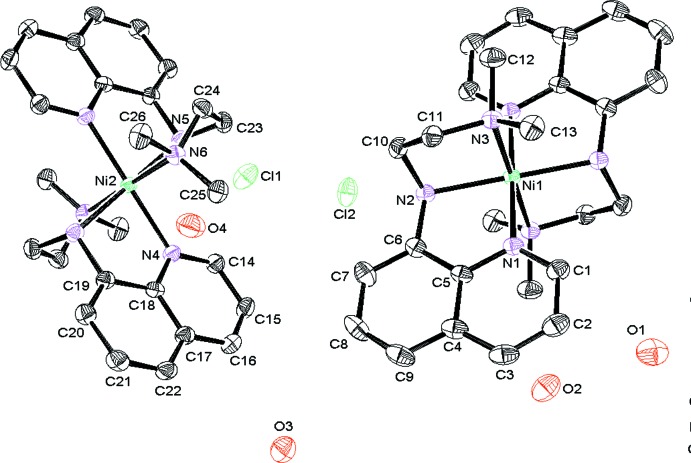
The asymmetric of the title complex showing atom labels and 50% probability displacement ellipsoids. Hydrogen atoms have been omitted.

. DOI: 10.1107/S1600536814019035/zs2312fig1.tif
The asymmetric of the title complex showing atom labels and 50% probability displacement ellipsoids. Hydrogen atoms have been omitted.

**Fig. 2.**
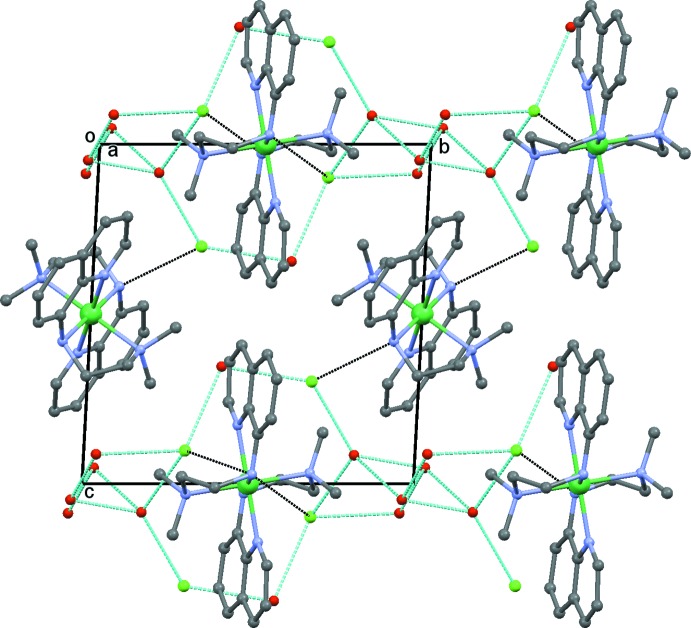
Packing in the crystal structure showing the O—H···O, O—H···Cl and N—H···Cl interactions as dotted lines.

. DOI: 10.1107/S1600536814019035/zs2312fig2.tif
Packing in the crystal structure showing the O—H⋯O, O—H⋯Cl and N—H⋯Cl inter­actions as dotted lines.

CCDC reference: 1020674

Additional supporting information:  crystallographic information; 3D view; checkCIF report

## Figures and Tables

**Table 1:** Hydrogen-bond geometry (Å, °)

*D*—H⋯*A*	*D*—H	H⋯*A*	*D*⋯*A*	*D*—H⋯*A*
N2—H2*A*⋯Cl2	1.00	2.27	3.2038 (18)	155
N5—H5⋯Cl1	1.00	2.33	3.2715 (19)	156
O1—H1*O*1⋯O2^i^	0.83 (1)	1.71 (1)	2.541 (5)	173 (6)
O1—H2*O*1⋯O2^ii^	0.84 (1)	2.05 (2)	2.863 (5)	165 (6)
O2—H1*O*2⋯Cl2^iii^	0.84 (1)	2.33 (1)	3.162 (3)	172 (4)
O2—H2*O*2⋯Cl1^iv^	0.83 (1)	2.37 (2)	3.179 (2)	163 (4)
O3—H1*O*3⋯Cl2^v^	0.85 (1)	2.38 (1)	3.222 (2)	174 (3)
O3—H2*O*3⋯Cl1^v^	0.85 (1)	2.36 (1)	3.2008 (19)	170 (3)
O4—H1*O*4⋯O1^vi^	0.85 (1)	1.91 (1)	2.755 (5)	177 (4)
O4—H2*O*4⋯Cl1	0.85 (1)	2.33 (1)	3.180 (3)	173 (3)

Symmetry codes: (i) 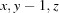; (ii) 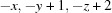; (iii) 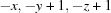; (iv) 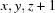; (v) 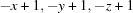; (vi) 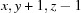.

## supplementary crystallographic information

### S1. Comment

As stated previously, (Al-Sudani & Kariuki, 2013), metal complexes of N-containing ligands occupy an important position in coordination chemistry (Singh *et al.*, 2008; Miodragovic *et al.*, 2008; Zhang *et al.*, 2009). Some quinoline-containing ligands show interesting biological activities (Puviarasan *et al.*, 2004). 8-[2-(dimethylamino)ethylamino]quinoline (NN'N"), is an asymmetric and potentially tridentate chelating ligand with the same donor atoms. With zinc (Al-Sudani, 2014) and cadmium ions (Al-Sudani & Kariuki, 2013), it forms neutral 1:1 metal to ligand mole ratio complexes with monomeric distorted square-pyrimidal and dimeric distorted octahedral geometries, respectively.

In the nickel(II) complex with the ligand 8-[2-(dimethylamino)ethylamino] quinoline, the title compound, C_26_H_34_N_6_Ni · 2Cl · 3.5(H_2_O), is ionic with a 1:2 metal to ligand mole ratio and has a slightly distorted bis-tridentate NiN_6_ octahedral coordination [Ni—N bond length range, 2.0777 (16)–2.2397 (17) Å] (Fig. 1). The asymmetric unit consists of two independent half molecules with the Ni^2+^ ions (Ni1 and Ni2) located on centres of inversion, as well as two chloride anions and 3.5 water molecules, one of which (O1) is disordered across an inversion centre. A network of O—H···O, O—H···Cl and N—H···Cl hydtrogen-bonding interactions are present in the crystal structure (Table 1) giving a three-dimensional network (Fig. 2). In the three complexes with this ligand (Zn, Cd and Ni), the unequivelant nitrogen donor atoms of the ligands are arranged facially around the metal ion.

### S2. Experimental

To a stirred methanoic solution (30 ml) containing a slight excess of the ligand (NN'N") (0.9 g; 0.0042 mol) kept under a positive nitrogen pressure, a methanoic solution (20 ml) of NiCl_2_ . 6H_2_O (0.47 g; 0.002 mol) was slowly added. The resulting brownish violet solution was stirred at room temperature overnight. A small amount of anhydrous MgSO_4_ was added and the reaction solution was stirred for a further one hour. After the removal of the drying agent by filtration, the solvent was removed by vacuum. The solid was washed twice with a small amount of diethyl ether (15 ml) to remove any of the unreacted ligand and was then dried under vacuum at 50 °C. The mass of the collected solid was 0.5 g which, based on the molecular formula of [Ni(NN'N")2] Cl_2_ represented a yield of *ca.* 45%. A suitable light brown–violet block shaped crystal of the title complex was obtained *via* slow diffusion of diethyl ether into a small amount of an acetonitrile solution of the compound kept under an atmosphere of nitrogen gas. Single crystal X-ray structure determination has identified the complex as [Ni(NN'N")2] Cl_2_·3.5H_2_O. In the process of measuring the melting point, the colour of the crystalline brown–violet solid changed to pale green and finally to very dark green. The dark green material was identified as a bimetallic complex of the formula [Ni(NN'N")Cl_2_]_2_ which decomposed at *ca.* 240 °C.

### S3. Refinement

H atoms were positioned geometrically (C—H = 0.95–0.99 Å and N—H = 1.00 Å) and refined using a riding model, with *U*_iso_(H) constrained to be 1.2 times *U*_eq_ of the bonded atom except for the methyl groups where it was 1.5 times with free rotation about the C—C bond. The geometry of the water molecules was constrained during refinement with O—H = 0.84 (2) Å and *U*_iso_(H) = 1.5 times *U*_eq_(O).

### Crystal data

**Table:**

[Ni(C_13_H_17_N_3_)_2_]Cl_2_·3.5H2O	*Z* = 2
*M**_r_* = 623.26	*F*(000) = 658
Triclinic, *P*1	*D*_x_ = 1.408 Mg m^−^^3^
*a* = 10.6940 (2) Å	Mo *K*α radiation, λ = 0.71073 Å
*b* = 11.8612 (4) Å	Cell parameters from 5592 reflections
*c* = 12.1088 (3) Å	θ = 2.0–25.2°
α = 90.520 (1)°	µ = 0.88 mm^−^^1^
β = 101.181 (2)°	*T* = 150 K
γ = 102.259 (2)°	Block, violet
*V* = 1470.39 (7) Å^3^	0.18 × 0.16 × 0.08 mm

### Data collection

**Table:**

Nonius KappaCCD diffractometer	6714 independent reflections
Radiation source: fine-focus sealed tube	5592 reflections with *I* > 2σ(*I*)
Graphite monochromator	*R*_int_ = 0.023
CCD slices, ω and φ scans	θ_max_ = 27.7°, θ_min_ = 2.0°
Absorption correction: multi-scan (*DENZO*/*SCALEPACK*; Otwinowski & Minor, 1997)	*h* = −13→13
*T*_min_ = 0.857, *T*_max_ = 0.933	*k* = −14→15
10258 measured reflections	*l* = −15→15

### Refinement

**Table:**

Refinement on *F*^2^	12 restraints
Least-squares matrix: full	Hydrogen site location: mixed
*R*[*F*^2^ > 2σ(*F*^2^)] = 0.039	H atoms treated by a mixture of independent and constrained refinement
*wR*(*F*^2^) = 0.090	*w* = 1/[σ^2^(*F*_o_^2^) + (0.0261*P*)^2^ + 1.4643*P*] where *P* = (*F*_o_^2^ + 2*F*_c_^2^)/3
*S* = 1.03	(Δ/σ)_max_ < 0.001
6714 reflections	Δρ_max_ = 0.46 e Å^−^^3^
383 parameters	Δρ_min_ = −0.59 e Å^−^^3^

### Special details

**Table:**

Geometry. All e.s.d.'s (except the e.s.d. in the dihedral angle between two l.s. planes) are estimated using the full covariance matrix. The cell e.s.d.'s are taken into account individually in the estimation of e.s.d.'s in distances, angles and torsion angles; correlations between e.s.d.'s in cell parameters are only used when they are defined by crystal symmetry. An approximate (isotropic) treatment of cell e.s.d.'s is used for estimating e.s.d.'s involving l.s. planes.

### Fractional atomic coordinates and isotropic or equivalent isotropic displacement parameters (Å^2^)

**Table:**

	*x*	*y*	*z*	*U*_iso_*/*U*_eq_	Occ. (<1)
C1	0.1605 (2)	−0.0934 (2)	0.70598 (18)	0.0262 (5)	
H1	0.0799	−0.1382	0.7180	0.031*	
C2	0.2745 (2)	−0.0947 (2)	0.78635 (19)	0.0335 (5)	
H2	0.2703	−0.1398	0.8505	0.040*	
C3	0.3911 (2)	−0.0301 (2)	0.7709 (2)	0.0336 (5)	
H3	0.4685	−0.0283	0.8255	0.040*	
C4	0.3964 (2)	0.0340 (2)	0.67356 (19)	0.0277 (5)	
C5	0.27726 (19)	0.02971 (18)	0.59635 (17)	0.0212 (4)	
C6	0.27629 (19)	0.09175 (18)	0.49678 (18)	0.0224 (4)	
C7	0.3910 (2)	0.1567 (2)	0.4765 (2)	0.0302 (5)	
H7	0.3908	0.1983	0.4098	0.036*	
C8	0.5094 (2)	0.1622 (2)	0.5540 (2)	0.0358 (6)	
H8	0.5881	0.2080	0.5391	0.043*	
C9	0.5123 (2)	0.1027 (2)	0.6499 (2)	0.0340 (5)	
H9	0.5928	0.1075	0.7012	0.041*	
C10	0.1447 (2)	0.00486 (19)	0.31787 (17)	0.0254 (4)	
H10A	0.2263	0.0272	0.2886	0.030*	
H10B	0.0709	0.0142	0.2578	0.030*	
C11	0.1257 (2)	−0.11934 (19)	0.34988 (18)	0.0248 (4)	
H11A	0.1214	−0.1698	0.2831	0.030*	
H11B	0.2010	−0.1288	0.4083	0.030*	
C12	−0.1075 (2)	−0.1898 (2)	0.29630 (19)	0.0314 (5)	
H12A	−0.0959	−0.2577	0.2559	0.047*	
H12B	−0.1096	−0.1262	0.2454	0.047*	
H12C	−0.1897	−0.2090	0.3233	0.047*	
C13	0.0092 (2)	−0.25829 (19)	0.45980 (19)	0.0298 (5)	
H13A	0.0202	−0.3208	0.4115	0.045*	
H13B	−0.0719	−0.2827	0.4879	0.045*	
H13C	0.0834	−0.2401	0.5237	0.045*	
C14	0.4792 (2)	0.57059 (19)	0.24055 (17)	0.0241 (4)	
H14	0.4000	0.5922	0.2081	0.029*	
C15	0.5159 (2)	0.5735 (2)	0.35907 (18)	0.0279 (5)	
H15	0.4634	0.5988	0.4049	0.034*	
C16	0.6272 (2)	0.5397 (2)	0.40723 (18)	0.0285 (5)	
H16	0.6525	0.5411	0.4870	0.034*	
C17	0.7048 (2)	0.50270 (19)	0.33842 (17)	0.0239 (4)	
C18	0.66485 (19)	0.50777 (17)	0.21999 (16)	0.0204 (4)	
C19	0.7417 (2)	0.47741 (18)	0.14687 (17)	0.0215 (4)	
C20	0.8527 (2)	0.43944 (19)	0.18998 (18)	0.0262 (5)	
H20	0.9051	0.4199	0.1409	0.031*	
C21	0.8896 (2)	0.4292 (2)	0.30789 (19)	0.0285 (5)	
H21	0.9649	0.4001	0.3369	0.034*	
C22	0.8183 (2)	0.4607 (2)	0.37999 (18)	0.0268 (5)	
H22	0.8451	0.4544	0.4588	0.032*	
C23	0.2283 (2)	0.4009 (2)	0.01160 (18)	0.0269 (5)	
H23A	0.1344	0.3883	−0.0234	0.032*	
H23B	0.2369	0.4071	0.0944	0.032*	
C24	0.2821 (2)	0.2997 (2)	−0.02055 (19)	0.0278 (5)	
H24A	0.2325	0.2276	0.0047	0.033*	
H24B	0.2705	0.2922	−0.1036	0.033*	
C25	0.4344 (2)	0.2817 (2)	0.15013 (18)	0.0282 (5)	
H25A	0.3904	0.2006	0.1522	0.042*	
H25B	0.3936	0.3307	0.1910	0.042*	
H25C	0.5269	0.2920	0.1856	0.042*	
C26	0.4790 (2)	0.2333 (2)	−0.0273 (2)	0.0321 (5)	
H26A	0.5716	0.2419	0.0070	0.048*	
H26B	0.4702	0.2500	−0.1072	0.048*	
H26C	0.4323	0.1539	−0.0203	0.048*	
Cl1	0.17945 (7)	0.69447 (6)	0.09796 (5)	0.04344 (16)	
Cl2	0.17828 (6)	0.32313 (5)	0.30115 (5)	0.03642 (14)	
N1	0.15965 (16)	−0.03312 (15)	0.61464 (14)	0.0205 (3)	
N2	0.15207 (16)	0.08100 (15)	0.41938 (14)	0.0203 (3)	
H2A	0.1392	0.1590	0.3958	0.024*	
N3	0.00298 (16)	−0.15441 (15)	0.39384 (14)	0.0216 (4)	
N4	0.54994 (16)	0.53923 (15)	0.17267 (14)	0.0211 (4)	
N5	0.30146 (16)	0.51031 (16)	−0.02755 (14)	0.0220 (4)	
H5	0.2917	0.5784	0.0167	0.026*	
N6	0.42293 (17)	0.31473 (15)	0.03107 (14)	0.0235 (4)	
Ni1	0.0000	0.0000	0.5000	0.01664 (9)	
Ni2	0.5000	0.5000	0.0000	0.01794 (9)	
O1	0.0938 (4)	0.0324 (3)	0.9528 (3)	0.0467 (9)	0.5
H1O1	0.059 (6)	−0.0378 (15)	0.943 (6)	0.070*	0.5
H2O1	0.055 (6)	0.065 (4)	0.992 (5)	0.070*	0.5
O2	0.0033 (3)	0.8165 (2)	0.91596 (19)	0.0614 (6)	
H1O2	−0.038 (4)	0.778 (3)	0.8572 (18)	0.092*	
H2O2	0.034 (4)	0.776 (3)	0.966 (2)	0.092*	
O3	0.78812 (19)	0.40276 (17)	0.65477 (15)	0.0400 (4)	
H1O3	0.794 (3)	0.4737 (10)	0.670 (2)	0.060*	
H2O3	0.804 (3)	0.374 (2)	0.7181 (14)	0.060*	
O4	0.2998 (2)	0.9624 (2)	0.0870 (2)	0.0639 (6)	
H1O4	0.238 (3)	0.987 (3)	0.046 (3)	0.096*	
H2O4	0.274 (4)	0.8898 (11)	0.093 (4)	0.096*	

### Atomic displacement parameters (Å^2^)

**Table:**

	*U*^11^	*U*^22^	*U*^33^	*U*^12^	*U*^13^	*U*^23^
C1	0.0267 (11)	0.0283 (12)	0.0244 (10)	0.0095 (9)	0.0033 (9)	0.0018 (9)
C2	0.0372 (13)	0.0379 (14)	0.0260 (11)	0.0161 (11)	−0.0010 (10)	0.0061 (10)
C3	0.0295 (12)	0.0382 (14)	0.0310 (12)	0.0154 (10)	−0.0074 (10)	−0.0024 (10)
C4	0.0236 (10)	0.0261 (12)	0.0328 (11)	0.0112 (9)	−0.0018 (9)	−0.0048 (9)
C5	0.0179 (9)	0.0204 (10)	0.0250 (10)	0.0067 (8)	0.0009 (8)	−0.0034 (8)
C6	0.0195 (10)	0.0204 (10)	0.0281 (10)	0.0074 (8)	0.0033 (8)	−0.0003 (8)
C7	0.0246 (11)	0.0251 (12)	0.0426 (13)	0.0062 (9)	0.0097 (10)	0.0040 (10)
C8	0.0190 (10)	0.0296 (13)	0.0577 (16)	0.0031 (9)	0.0074 (10)	−0.0013 (11)
C9	0.0198 (10)	0.0329 (13)	0.0470 (14)	0.0097 (9)	−0.0028 (10)	−0.0054 (11)
C10	0.0273 (11)	0.0286 (12)	0.0221 (10)	0.0071 (9)	0.0085 (8)	0.0006 (9)
C11	0.0247 (10)	0.0276 (12)	0.0245 (10)	0.0089 (9)	0.0073 (8)	−0.0033 (9)
C12	0.0305 (12)	0.0312 (13)	0.0302 (12)	0.0068 (10)	0.0007 (9)	−0.0094 (10)
C13	0.0395 (13)	0.0204 (11)	0.0320 (12)	0.0101 (9)	0.0095 (10)	−0.0006 (9)
C14	0.0229 (10)	0.0261 (11)	0.0249 (10)	0.0083 (9)	0.0058 (8)	0.0012 (9)
C15	0.0315 (12)	0.0333 (13)	0.0224 (10)	0.0100 (10)	0.0104 (9)	0.0000 (9)
C16	0.0331 (12)	0.0327 (13)	0.0198 (10)	0.0073 (10)	0.0053 (9)	0.0009 (9)
C17	0.0251 (10)	0.0234 (11)	0.0220 (10)	0.0046 (8)	0.0024 (8)	0.0027 (8)
C18	0.0199 (9)	0.0187 (10)	0.0203 (9)	0.0025 (8)	0.0003 (8)	0.0015 (8)
C19	0.0225 (10)	0.0205 (10)	0.0210 (10)	0.0045 (8)	0.0032 (8)	0.0025 (8)
C20	0.0250 (11)	0.0266 (11)	0.0278 (11)	0.0075 (9)	0.0049 (9)	0.0039 (9)
C21	0.0234 (11)	0.0312 (12)	0.0311 (11)	0.0105 (9)	0.0003 (9)	0.0074 (9)
C22	0.0273 (11)	0.0297 (12)	0.0207 (10)	0.0057 (9)	−0.0012 (8)	0.0062 (9)
C23	0.0221 (10)	0.0345 (13)	0.0253 (10)	0.0069 (9)	0.0065 (8)	0.0096 (9)
C24	0.0245 (11)	0.0268 (12)	0.0287 (11)	0.0002 (9)	0.0027 (9)	0.0039 (9)
C25	0.0331 (12)	0.0265 (12)	0.0268 (11)	0.0087 (9)	0.0074 (9)	0.0078 (9)
C26	0.0436 (14)	0.0224 (12)	0.0353 (12)	0.0120 (10)	0.0143 (11)	0.0018 (9)
Cl1	0.0589 (4)	0.0479 (4)	0.0332 (3)	0.0311 (3)	0.0112 (3)	0.0026 (3)
Cl2	0.0428 (3)	0.0371 (3)	0.0393 (3)	0.0192 (3)	0.0201 (3)	0.0156 (3)
N1	0.0195 (8)	0.0222 (9)	0.0202 (8)	0.0077 (7)	0.0013 (7)	−0.0009 (7)
N2	0.0191 (8)	0.0208 (9)	0.0216 (8)	0.0067 (7)	0.0029 (7)	0.0015 (7)
N3	0.0221 (8)	0.0204 (9)	0.0223 (8)	0.0060 (7)	0.0031 (7)	−0.0016 (7)
N4	0.0222 (8)	0.0229 (9)	0.0183 (8)	0.0057 (7)	0.0032 (7)	0.0013 (7)
N5	0.0224 (8)	0.0249 (9)	0.0196 (8)	0.0075 (7)	0.0037 (7)	0.0026 (7)
N6	0.0257 (9)	0.0212 (9)	0.0235 (9)	0.0062 (7)	0.0039 (7)	0.0035 (7)
Ni1	0.01521 (17)	0.01787 (19)	0.01697 (17)	0.00518 (13)	0.00185 (13)	0.00047 (13)
Ni2	0.01852 (18)	0.02006 (19)	0.01495 (17)	0.00518 (14)	0.00156 (13)	0.00111 (14)
O1	0.056 (2)	0.028 (2)	0.051 (2)	0.0087 (18)	0.0000 (19)	−0.0011 (17)
O2	0.0768 (16)	0.0634 (15)	0.0489 (12)	0.0309 (13)	0.0067 (11)	0.0157 (11)
O3	0.0440 (10)	0.0422 (11)	0.0364 (9)	0.0131 (9)	0.0102 (8)	0.0017 (8)
O4	0.0583 (14)	0.0596 (15)	0.0636 (15)	0.0000 (12)	0.0018 (11)	−0.0004 (12)

### Geometric parameters (Å, º)

**Table:**

C1—N1	1.322 (3)	C19—N5^i^	1.448 (3)
C1—C2	1.407 (3)	C20—C21	1.419 (3)
C1—H1	0.9500	C20—H20	0.9500
C2—C3	1.365 (3)	C21—C22	1.362 (3)
C2—H2	0.9500	C21—H21	0.9500
C3—C4	1.412 (3)	C22—H22	0.9500
C3—H3	0.9500	C23—N5	1.499 (3)
C4—C9	1.412 (3)	C23—C24	1.515 (3)
C4—C5	1.418 (3)	C23—H23A	0.9900
C5—N1	1.378 (3)	C23—H23B	0.9900
C5—C6	1.418 (3)	C24—N6	1.486 (3)
C6—C7	1.370 (3)	C24—H24A	0.9900
C6—N2	1.450 (3)	C24—H24B	0.9900
C7—C8	1.411 (3)	C25—N6	1.486 (3)
C7—H7	0.9500	C25—H25A	0.9800
C8—C9	1.364 (4)	C25—H25B	0.9800
C8—H8	0.9500	C25—H25C	0.9800
C9—H9	0.9500	C26—N6	1.480 (3)
C10—N2	1.498 (3)	C26—H26A	0.9800
C10—C11	1.508 (3)	C26—H26B	0.9800
C10—H10A	0.9900	C26—H26C	0.9800
C10—H10B	0.9900	N1—Ni1	2.0909 (17)
C11—N3	1.491 (3)	N2—Ni1	2.1189 (16)
C11—H11A	0.9900	N2—H2A	1.0000
C11—H11B	0.9900	N3—Ni1	2.2374 (17)
C12—N3	1.485 (3)	N4—Ni2	2.0778 (16)
C12—H12A	0.9800	N5—C19^i^	1.448 (3)
C12—H12B	0.9800	N5—Ni2	2.1143 (17)
C12—H12C	0.9800	N5—H5	1.0000
C13—N3	1.481 (3)	N6—Ni2	2.2397 (17)
C13—H13A	0.9800	Ni1—N1^ii^	2.0909 (17)
C13—H13B	0.9800	Ni1—N2^ii^	2.1189 (16)
C13—H13C	0.9800	Ni1—N3^ii^	2.2373 (17)
C14—N4	1.319 (3)	Ni2—N4^i^	2.0777 (16)
C14—C15	1.411 (3)	Ni2—N5^i^	2.1144 (17)
C14—H14	0.9500	Ni2—N6^i^	2.2397 (17)
C15—C16	1.361 (3)	O1—H1O1	0.833 (10)
C15—H15	0.9500	O1—H2O1	0.836 (10)
C16—C17	1.411 (3)	O2—H1O2	0.835 (10)
C16—H16	0.9500	O2—H2O2	0.833 (10)
C17—C22	1.414 (3)	O3—H1O3	0.846 (10)
C17—C18	1.420 (3)	O3—H2O3	0.846 (10)
C18—N4	1.380 (3)	O4—H1O4	0.848 (10)
C18—C19	1.411 (3)	O4—H2O4	0.854 (10)
C19—C20	1.370 (3)		
			
N1—C1—C2	123.5 (2)	H23A—C23—H23B	108.2
N1—C1—H1	118.2	N6—C24—C23	111.42 (18)
C2—C1—H1	118.2	N6—C24—H24A	109.3
C3—C2—C1	119.1 (2)	C23—C24—H24A	109.3
C3—C2—H2	120.5	N6—C24—H24B	109.3
C1—C2—H2	120.5	C23—C24—H24B	109.3
C2—C3—C4	119.9 (2)	H24A—C24—H24B	108.0
C2—C3—H3	120.1	N6—C25—H25A	109.5
C4—C3—H3	120.1	N6—C25—H25B	109.5
C9—C4—C3	124.0 (2)	H25A—C25—H25B	109.5
C9—C4—C5	118.6 (2)	N6—C25—H25C	109.5
C3—C4—C5	117.4 (2)	H25A—C25—H25C	109.5
N1—C5—C6	117.79 (18)	H25B—C25—H25C	109.5
N1—C5—C4	122.12 (19)	N6—C26—H26A	109.5
C6—C5—C4	120.09 (19)	N6—C26—H26B	109.5
C7—C6—C5	119.51 (19)	H26A—C26—H26B	109.5
C7—C6—N2	123.0 (2)	N6—C26—H26C	109.5
C5—C6—N2	117.51 (17)	H26A—C26—H26C	109.5
C6—C7—C8	120.5 (2)	H26B—C26—H26C	109.5
C6—C7—H7	119.8	C1—N1—C5	117.98 (18)
C8—C7—H7	119.8	C1—N1—Ni1	128.92 (14)
C9—C8—C7	120.8 (2)	C5—N1—Ni1	112.58 (13)
C9—C8—H8	119.6	C6—N2—C10	110.88 (16)
C7—C8—H8	119.6	C6—N2—Ni1	109.03 (12)
C8—C9—C4	120.5 (2)	C10—N2—Ni1	106.62 (12)
C8—C9—H9	119.7	C6—N2—H2A	110.1
C4—C9—H9	119.7	C10—N2—H2A	110.1
N2—C10—C11	109.39 (17)	Ni1—N2—H2A	110.1
N2—C10—H10A	109.8	C13—N3—C12	106.35 (17)
C11—C10—H10A	109.8	C13—N3—C11	108.99 (16)
N2—C10—H10B	109.8	C12—N3—C11	108.26 (16)
C11—C10—H10B	109.8	C13—N3—Ni1	112.67 (13)
H10A—C10—H10B	108.2	C12—N3—Ni1	116.48 (13)
N3—C11—C10	110.47 (17)	C11—N3—Ni1	103.85 (12)
N3—C11—H11A	109.6	C14—N4—C18	118.37 (17)
C10—C11—H11A	109.6	C14—N4—Ni2	129.17 (14)
N3—C11—H11B	109.6	C18—N4—Ni2	111.46 (13)
C10—C11—H11B	109.6	C19^i^—N5—C23	111.93 (16)
H11A—C11—H11B	108.1	C19^i^—N5—Ni2	107.77 (12)
N3—C12—H12A	109.5	C23—N5—Ni2	106.88 (12)
N3—C12—H12B	109.5	C19^i^—N5—H5	110.1
H12A—C12—H12B	109.5	C23—N5—H5	110.1
N3—C12—H12C	109.5	Ni2—N5—H5	110.1
H12A—C12—H12C	109.5	C26—N6—C25	106.69 (17)
H12B—C12—H12C	109.5	C26—N6—C24	108.98 (17)
N3—C13—H13A	109.5	C25—N6—C24	108.49 (16)
N3—C13—H13B	109.5	C26—N6—Ni2	113.21 (13)
H13A—C13—H13B	109.5	C25—N6—Ni2	117.61 (13)
N3—C13—H13C	109.5	C24—N6—Ni2	101.49 (13)
H13A—C13—H13C	109.5	N1^ii^—Ni1—N1	180.00 (9)
H13B—C13—H13C	109.5	N1^ii^—Ni1—N2	99.10 (6)
N4—C14—C15	123.0 (2)	N1—Ni1—N2	80.90 (6)
N4—C14—H14	118.5	N1^ii^—Ni1—N2^ii^	80.90 (6)
C15—C14—H14	118.5	N1—Ni1—N2^ii^	99.11 (6)
C16—C15—C14	119.5 (2)	N2—Ni1—N2^ii^	180.00 (7)
C16—C15—H15	120.3	N1^ii^—Ni1—N3^ii^	88.88 (6)
C14—C15—H15	120.3	N1—Ni1—N3^ii^	91.12 (6)
C15—C16—C17	119.85 (19)	N2—Ni1—N3^ii^	97.02 (6)
C15—C16—H16	120.1	N2^ii^—Ni1—N3^ii^	82.98 (6)
C17—C16—H16	120.1	N1^ii^—Ni1—N3	91.12 (6)
C16—C17—C22	124.18 (19)	N1—Ni1—N3	88.88 (6)
C16—C17—C18	117.32 (19)	N2—Ni1—N3	82.98 (6)
C22—C17—C18	118.50 (19)	N2^ii^—Ni1—N3	97.02 (6)
N4—C18—C19	118.02 (17)	N3^ii^—Ni1—N3	180.0
N4—C18—C17	121.87 (18)	N4^i^—Ni2—N4	180.0
C19—C18—C17	120.10 (19)	N4^i^—Ni2—N5	81.04 (6)
C20—C19—C18	119.92 (19)	N4—Ni2—N5	98.96 (6)
C20—C19—N5^i^	122.84 (18)	N4^i^—Ni2—N5^i^	98.96 (6)
C18—C19—N5^i^	117.24 (18)	N4—Ni2—N5^i^	81.04 (6)
C19—C20—C21	120.1 (2)	N5—Ni2—N5^i^	180.0
C19—C20—H20	120.0	N4^i^—Ni2—N6	89.87 (6)
C21—C20—H20	120.0	N4—Ni2—N6	90.13 (6)
C22—C21—C20	120.8 (2)	N5—Ni2—N6	84.17 (7)
C22—C21—H21	119.6	N5^i^—Ni2—N6	95.83 (7)
C20—C21—H21	119.6	N4^i^—Ni2—N6^i^	90.13 (6)
C21—C22—C17	120.5 (2)	N4—Ni2—N6^i^	89.87 (6)
C21—C22—H22	119.7	N5—Ni2—N6^i^	95.83 (7)
C17—C22—H22	119.7	N5^i^—Ni2—N6^i^	84.17 (7)
N5—C23—C24	109.85 (17)	N6—Ni2—N6^i^	180.00 (4)
N5—C23—H23A	109.7	H1O1—O1—H2O1	110 (3)
C24—C23—H23A	109.7	H1O2—O2—H2O2	113 (2)
N5—C23—H23B	109.7	H1O3—O3—H2O3	105 (2)
C24—C23—H23B	109.7	H1O4—O4—H2O4	108 (2)

Symmetry codes: (i) −*x*+1, −*y*+1, −*z*; (ii) −*x*, −*y*, −*z*+1.

### Hydrogen-bond geometry (Å, º)

**Table:**

*D*—H···*A*	*D*—H	H···*A*	*D*···*A*	*D*—H···*A*
N2—H2*A*···Cl2	1.00	2.27	3.2038 (18)	155
N5—H5···Cl1	1.00	2.33	3.2715 (19)	156
O1—H1*O*1···O2^iii^	0.83 (1)	1.71 (1)	2.541 (5)	173 (6)
O1—H2*O*1···O2^iv^	0.84 (1)	2.05 (2)	2.863 (5)	165 (6)
O2—H1*O*2···Cl2^v^	0.84 (1)	2.33 (1)	3.162 (3)	172 (4)
O2—H2*O*2···Cl1^vi^	0.83 (1)	2.37 (2)	3.179 (2)	163 (4)
O3—H1*O*3···Cl2^vii^	0.85 (1)	2.38 (1)	3.222 (2)	174 (3)
O3—H2*O*3···Cl1^vii^	0.85 (1)	2.36 (1)	3.2008 (19)	170 (3)
O4—H1*O*4···O1^viii^	0.85 (1)	1.91 (1)	2.755 (5)	177 (4)
O4—H2*O*4···Cl1	0.85 (1)	2.33 (1)	3.180 (3)	173 (3)

Symmetry codes: (iii) *x*, *y*−1, *z*; (iv) −*x*, −*y*+1, −*z*+2; (v) −*x*, −*y*+1, −*z*+1; (vi) *x*, *y*, *z*+1; (vii) −*x*+1, −*y*+1, −*z*+1; (viii) *x*, *y*+1, *z*−1.
