# Supplementary figures and images for: Crystal structure of bis­{N-[2-(di­methyl­amino)­eth­yl]quinolin-8-amine-κ3 N,N′,N′′}nickel(II) dichloride 3.5-hydrate
Source: Acta Crystallogr Sect E Struct Rep Online. 2014 Aug 30;70(Pt 9):m339–40. doi: 10.1107/S1600536814019035 (PMC4186116; doi:10.1107/S1600536814019035)

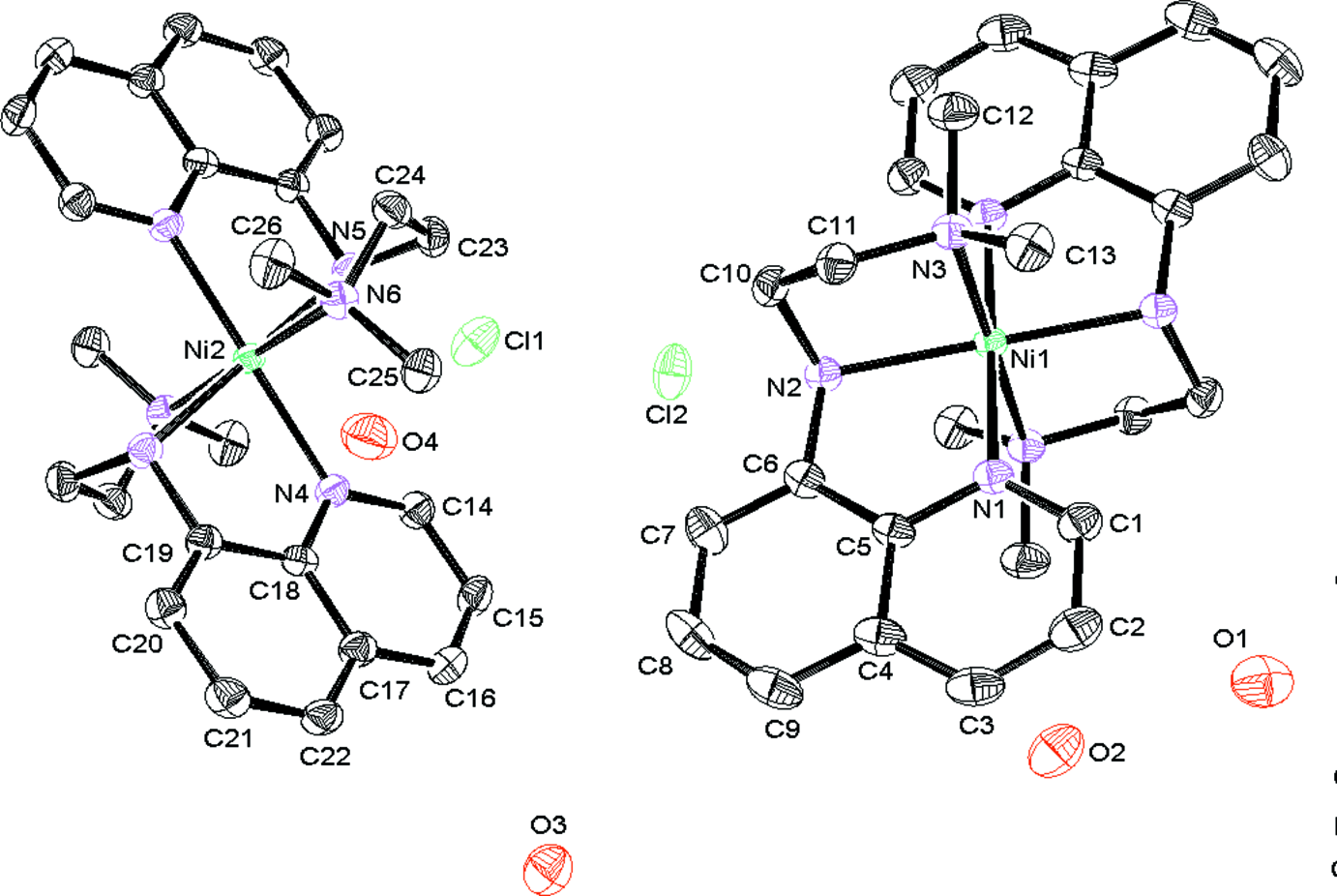

Supplement: Supplementary file 3 [file e-70-0m339-fig1.tif]

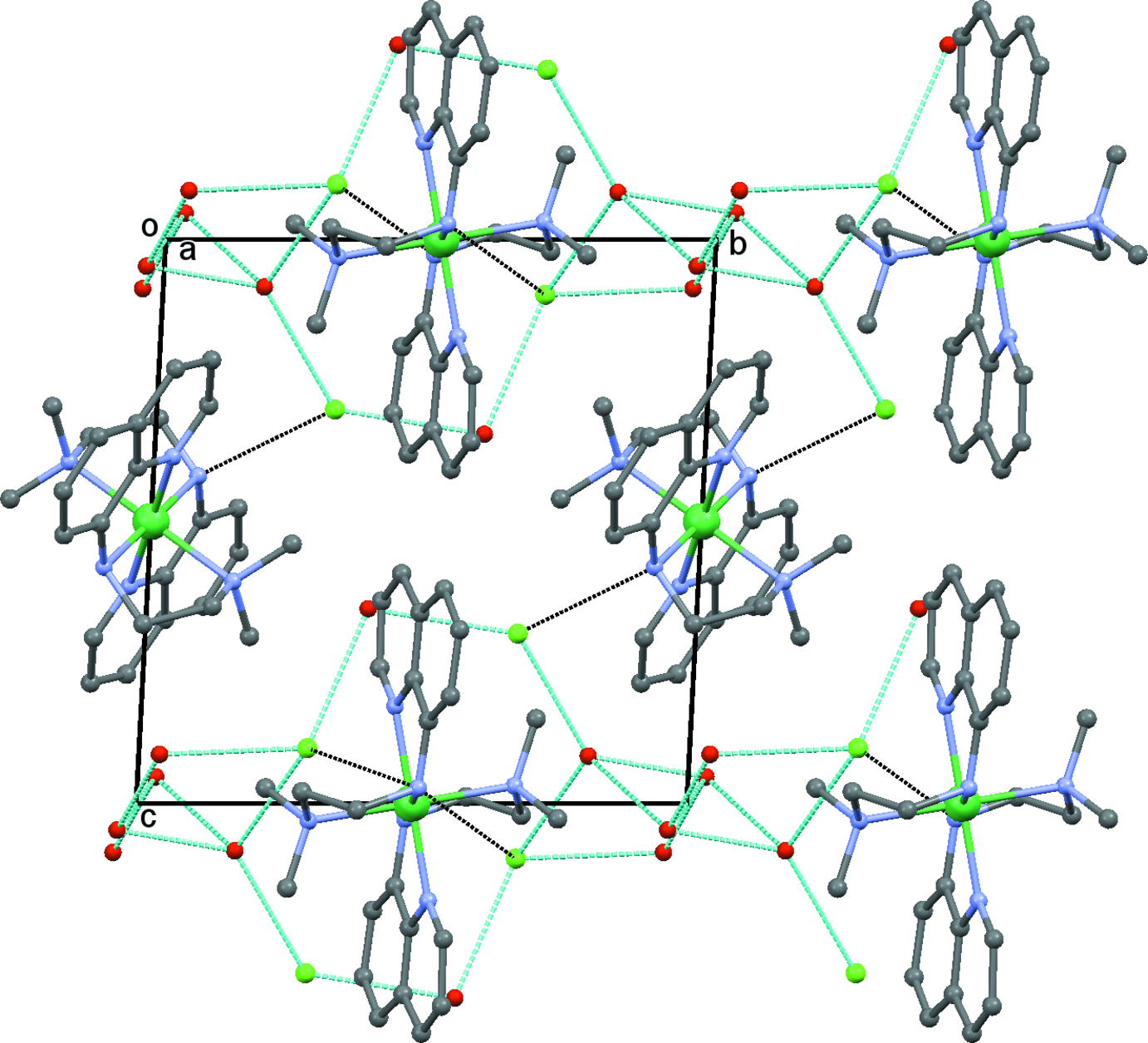

Supplement: Supplementary file 4 [file e-70-0m339-fig2.tif]
